# Individual and community-level factors of availability of observed water, soap, and other cleansing agents for hand washing practice in Ethiopia: a multilevel mixed-effects analysis of the 2021 performance monitoring for action Ethiopia

**DOI:** 10.3389/fpubh.2024.1418379

**Published:** 2024-07-22

**Authors:** Natnael Kebede, Amare Mebrat Delie, Eyob Ketema Bogale, Tadele Fentabel Anagaw, Eneyew Talie Fenta, Ousman Adal

**Affiliations:** ^1^Department of Health Promotion, School of Public Health, College of Medicine and Health Sciences, Wollo University, Dessie, Ethiopia; ^2^Department of Public Health, College of Medicine and Health Sciences, Injibara University, Injibara, Ethiopia; ^3^Department of Health Promotion and Behavioral Science, College of Medicine and Health Sciences, Bahir Dar University, Bahir Dar, Ethiopia; ^4^Department of Emergency Nurse, College of Medicine and Health Sciences, Bahir Dar University, Bahir Dar, Ethiopia

**Keywords:** hand washing practice, factors, multilevel analysis, Ethiopia, predictors

## Abstract

**Introduction:**

This study delves into individual and community-level factors influencing the availability of water, soap, and cleansing agents for handwashing in Ethiopia. Its comprehensive exploration offers nuanced insights, informing targeted interventions and policies to effectively enhance handwashing resources across Ethiopia. Therefore, this study aimed to assess individual and community-level factors of availability of observed water, soap, and other cleansing agents for hand washing practices in Ethiopia.

**Methods:**

Data from the 2021 PMA-ET, encompassing 24,747 household participants, informed this study. Employing STATA version 17.0, a multi-level mixed-effect logistic regression analysis was performed to identify individual and community-level factors. Adjusted odds ratios with a 95% confidence interval conveyed the strength and direction of associations, with significance determined at *p* < 0.05.

**Results:**

Significant factors affecting handwashing resources availability: water, soap, and cleansing agents included education status such as Participants aged below 25 and between 25 and 64 (OR = 1.38; 95% CI: 1.0891, 1.7631) and (OR = 1.45; 95% CI: 1.1431, 1.8621) respectively, individuals with no formal education and those with only primary education were 40 and 39% less likely (OR = 0.60; 95% CI: 0. 47,191, 0. 77,317) and (OR = 0.61; 95% CI: 0. 46,526, 0. 80,124) respectively, those who had poor and had middle wealth status were (OR = 0.30; 95% CI: 0. 24,955, 0.37165) and (OR = 0.37; 95% CI: 0.31465, 0. 44,973) respectively, who had media exposure (OR = 2.88; 95% CI: 2.5565, 3.2615), water sources, various sources like Piped Water, tube well, dug well, spring, rainwater, and surface water were less likely to provide access compared to bottled water. Furthermore, clusters with a lower proportion of primary education [AOR = 0.13, 95% CI: (0.04303, 0.44515)], and those with a higher proportion of middle wealth status [AOR = 3.26, 95% CI: (1.071, 9.9245)].

**Conclusion:**

The study uncovered individual and community-level factors impacting the availability of water, soap, and cleansing agents for handwashing in Ethiopia. Individual factors like age, education, wealth, water source, media exposure, Community factors such as education levels and wealth status showed significant associations. Community initiatives should boost primary education and equitable wealth distribution to ensure widespread access to hand-washing resources, fostering improved hygiene practices.

## Introduction

The term availability of observed water, soap, and other cleansing agents for hand washing practice refers to the accessibility and presence of essential resources necessary for maintaining proper hand hygiene. It encompasses the physical presence of water sources, soap, and other cleaning agents that can be visibly observed in a given environment, indicating the potential for individuals to engage in effective hand-washing practices (1–3). The availability of observed water, soap, and other cleansing agents for hand washing practices is a critical factor in promoting good hygiene practices and preventing the spread of infectious diseases. Globally, it is estimated that only 60% of the population has access to basic hand-washing facilities with soap and water (4), while in Africa, this figure drops to just 15% (5). In Ethiopia, only 34% of the population has access to basic hand-washing facilities with soap and water (6).

The unavailability of observed water, soap, and other cleansing agents for hand washing practices can have serious consequences on public health. Hand washing is one of the most effective ways to prevent the spread of infectious diseases and consequence of infectious disease, such as diarrhea, cholera, and respiratory infections. When people do not have access to clean water and soap, they are more likely to use contaminated water or not wash their hands at all, increasing the risk of transmitting diseases. This can lead to an increase in morbidity and mortality rates, especially among vulnerable populations such as children, pregnant women, and the older adult. Additionally, it can also have economic consequences as it can lead to increased healthcare costs and reduced productivity due to illness (7–9).

Previous studies have indicated different factors associated with hand-washing practices. Age, educational status, wealth status, water source, family size, media exposure, and related factors have been found to influence hand-washing practices (4, 7, 10–12).

This study addresses a critical gap by investigating both individual and community-level factors of availability of observed water, soap, and other cleansing agents for hand washing practice in Ethiopia, using a Multilevel Mixed-Effects Analysis of the 2021 PMA-ET. practiceThe added value lies in informing targeted, context-specific interventions to improve hand hygiene, thereby enhancing public health outcomes and ensuring equitable access to handwashing facilities across diverse communities.practice.

## Materials and methods

### Study area and data source

In the PMA-ET 2021 study, a community-based cross-sectional design employed a two-stage cluster approach with residential areas (urban and rural) and sub-regions as strata, ensuring representation across all 11 geographic regions in Ethiopia. Notably, 95% of the target population, where households are selected in sampled clusters, resides in four key regions: Addis Ababa, Amhara, Oromia, and South Nation Nationalities of People (SNNP). To address regions with less than 5% of the target population, a sixth synthetic region, denoted as “other,” was created. Due to population distribution and resource constraints, regional representative samples were taken exclusively in the four major regions. The sampling design, comprising 321 Enumeration Areas, aims to achieve a national-level margin of error below 2%, below 3% for urban and rural estimates, and below 5% at each of the four regional levels, ensuring robust and precise estimates of availability of observed water, soap, and other cleansing agents for hand washing practice in Ethiopia. The secondary data for this analysis were obtained from PMA-ET of 2021 which was found in the PMA portal (pmadata.org).

### Population

The study population comprised all households in Ethiopia. A weighted total of 24,747 participants in households were included in the analysis, encompassing all variables of interest. Households with missing information regarding the availability of observed water, soap, and other cleansing agents for hand washing practice were deliberately excluded from the study’s analysis. This exclusion specifically targeted households from enumeration areas where geographic coordinates were recorded as zero, ensuring that only comprehensive data sets were considered for accurate assessment.practice.

### Variable measurement

The dependent variable of the availability of observed water, soap, and other cleansing agents for hand washing practice was classified dichotomously as “Yes/No.” The availability of observed water, soap, and other cleansing agents during the time of the interview was categorized as “Yes” and no availability of observed water, soap, and other cleansing agents during the time of the interview was categorized as “No.”

#### Individual-level variables

Age, sex of participants, education status (labeled as no education, primary, secondary, and higher education), wealth index (labeled as poor, middle, and rich), Media exposure- exposure to mass media (labeled as Yes or No), Family size (small less than 5 and high greater than 5), and water source.

#### Community-level variables

While region and place of residence were sourced directly as community-level variables from the 2021 PMA-ET data, the remaining variables were not available in that form. Consequently, these variables were aggregated at the community level based on individual information. The aggregated values were then categorized as low or high, with classifications determined by whether the median values or proportions of the clusters fell below or above the national level. As a result, community-level educational status, community-level wealth status, and community-level media exposure were established as community-level variables in the analysis.

### Data quality control and analysis

Consistency and completeness were assured through preliminary data cleaning before recording, labeling, and conducting exploratory analysis using Stata/SE version 17.0. The presentation of frequency distributions in tables and text, along with the estimation of proportions and frequencies, followed the application of sample weights. This adjustment was made to account for the non-proportional allocation of the sample across different regions, urban and rural areas, and potential variations in response rates, aligning with the guidelines of PMA-ET.

After confirming the data’s eligibility for multilevel analysis, as evidenced by an Intra-cluster Correlation Coefficient (ICC) exceeding 10% (specifically, ICC = 88.4%), a multilevel analysis was conducted (13). Given the hierarchical structure of PMA data, wherein individuals (level 1) are nested within communities (level 2), a two-level mixed-effects logistic regression model was applied. This model aimed to estimate both the independent (fixed) effects of explanatory variables and the community-level random effects on the availability of observed water, soap, and other cleansing agents for hand washing practice. The log of the probability of availability of observed water, soap, and other cleansing agents for hand washing practice was modeled using a two-level multilevel approach. Four models were constructed in this analysis.

#### Empty model

Executing this model without including any factors aimed to assess the random effect of between-cluster variability. The estimation of the intra-class correlation coefficient (ICC), derived from both the between-cluster and within-cluster variability, was conducted to ascertain whether the data justified employing a multilevel approach for analyses. The ICC provides insight into the magnitude of between-cluster variability.

#### Individual-level factors model

The second model investigated the impact of individual characteristics on the availability of observed water, soap, and other cleansing agents for hand washing practice. Additionally, the intra-class correlation coefficient (ICC) was computed to observe whether there was a reduction in between-cluster variability when individual factors were introduced into the empty model.

#### Community-level factors model

In this model, only cluster characteristics were considered, excluding individual characteristics. The unit of analysis for this model was the cluster.

#### Combined model

In this model, the significant features of individuals at the household level and clusters were simultaneously incorporated to unveil their combined fixed and random effects.

Multicollinearity among the exposure variables was assessed using the variance inflation factor (VIF), and all VIF values were found to be below 7.5 (14). The study conveyed the impact of individual and community-level factors on the availability of observed water, soap, and other cleansing agents for hand washing using Odds Ratios (OR) for fixed effects. Additionally, community-level effects were estimated with their corresponding 95% Confidence Intervals (95% CI) practice.

The random effects were measured by the intra-class correlation coefficient (ICC), median odds ratio (MOR), and proportional change in variance (PCV). The Intra-class Correlation Coefficient (ICC) quantifies the extent of variation in the availability of observed water, soap, and other cleansing agents for hand washing practice in Ethiopia attributable to community characteristics. A higher ICC indicates greater significance of community characteristics in comprehending individual variations in the availability of observed water, soap, and other cleansing agents for hand washing practice in Ethiopia.

**The Median Odds Ratio (MOR)** is characterized as the median value of the odds ratio between the area at the highest risk and the area at the lowest risk when randomly selecting two areas. In the context of this study, MOR illustrates the degree to which the individual likelihood of availability of observed water, soap, and other cleansing agents for hand washing practice in Ethiopia is influenced by their residential area. Proportional change in variance (PCV) gauges the overall variation attributed to both individual-level and area-level factors within the multilevel model.

#### Model fit statistics

To evaluate the goodness of fit and guide the selection of nested models (individual- and community-level models), Akaike’s Information Criterion (AIC) and Schwarz’s Bayesian Information Criterion (BIC) were employed. The AIC and BIC values were compared across successive models, and the model exhibiting the lowest value was deemed the most fitting (15, 16).

## Results

### Socio-demographic characteristics of respondents

A total of 24,747 members in the households were included in the study. The majority (58.35%) of participants in urban areas were found to be aged less than 25 years old, while nearly half (50.46%) were females, with almost the majority (82.28%) having no formal education. In terms of wealth index, approximately 42.01% of participants were classified as having a rich wealth status. Moreover, regarding water sources, 44.07% of urban participants had access to piped water. Family size varied, with 59.56% of participants having more than 5 members. Additionally, media exposure was prevalent among 51.98% of participants. The unit of analysis for the characteristics of community-level factors was the cluster. For this study, the findings included a cluster of 221 households. Most 23,959 (96.81%) were from developed regions and more than two-thirds resided in rural areas (70.99%). The current study attempted to form community-level factors by aggregating values of different individual characteristics. More than half of the cluster belongs to communities with a lower proportion of higher education (56.32%). Similarly, about 59.35% of the clusters had a Lower proportion of media exposure and 60.45% of the clusters had a higher proportion of poor wealth status (Table 1).

**Table 1:** Socio-demographic characteristics of respondents, 2021 performance monitoring for action Ethiopia (PMA-ET).

Variables	Category	Weighted frequency	Weighted *n*%
Age	<25	14,439	58.35
	25–64	9,172	37.06
	>65	1,135	4.59
Sex	Male	12,259	49.54
	Female	12,489	50.46
Educational status	no educated	20,360	82.28
	primary education	2,333	9.42
	secondary education	1,388	5.61
	higher education	666	2.69
Wealth status	Poor	9,423	38.08
	Middle	4,928	19.91
	Rich	10,396	42.01
Water source	Piped Water	10,907	44.07
	tube well	3,323	13.43
	dug well	1,657	6.70
	Spring	7,264	29.35
	Rainwater	161	0.65
	surface water	1,086	4.39
	bottled water	349	1.41
Family size	<=5	10,007	40.44
	>5	|14,740	59.56
Media exposure	No	12,863	51.98
	Yes	11,884	48.02

**Region**		
Emerging regions	788	3.19
Developed regions	23,959	96.81
**Residence**		
Urban	7,180	29.01
Rural	17,567	70.99
**Community educational status**		
A lower proportion of higher education	13,898	56.32
A higher proportion of higher education	10,779	43.68
**Community wealth status**		
A lower proportion of poor	9,760	39.55
A higher proportion of poor	14,917	60.45
**Community media exposure**		
A lower proportion of media exposure	14,646	59.35
A higher proportion of media exposure	10,031	40.65

### Availability of observed water, soap, and other cleansing agents for hand washing practice

The proportion of availability of observed water, soap, and other cleansing agents for hand washing practice was 21.7% [95% CI: (21.2, 22.2)].

### Multi-level mixed effect logistic regression of availability of observed water, soap, and other cleansing agents for hand washing practice

#### Multilevel analysis (fixed effect analysis)

In the fully adjusted model, accounting for both individual and community-level factors, factors such as age, educational level, household wealth status, media exposure, water source, community educational status, and community-level wealth status were factors associated with availability of observed water, soap, and other cleansing agents for hand washing practice in Ethiopia.

### Individual and community-level factors associated with availability of observed water, soap, and other cleansing agents for hand washing practice

Participants aged below 25 and between 25 and 64 had 1.38 times (OR = 1.38; 95% CI: 1.0891, 1.7631) and 1.45 times (OR = 1.45; 95% CI: 1.1431, 1.8621) higher availability of observed water, soap, and other cleansing agents for hand washing practice compared to those aged 65 and above, respectively.Practice. Notably, Individuals with no formal education and those with only primary education were 40% (OR = 0.60; 95% CI: 0.47191, 0.77317) and 39% (OR = 0.61; 95% CI: 0.46526, 0.80124) less likely to availability of observed water, soap, and other cleansing agents for hand washing practice compared to those with higher education, respectively. Likewise, those with poor and middle wealth statuses were 70% (OR = 0.30; 95% CI: 0.24955, 0.37165) and 63% (OR = 0.37; 95% CI: 0.31465, 0.44973) less likely to availability to observed water, soap, and other cleansing agents for hand washing practice compared to those with rich wealth status, respectivelyPractice The odds of availability of observed water, soap, and other cleansing agents for hand washing Practice in Ethiopia who had media exposure were 2.88 more likely l (OR = 2.88; 95% CI: 2.5565, 3.2615) as compared with those who had no media exposure. Regarding water sources, various sources like Piped Water, tube wells, dug wells, springs, rainwater, and surface water were less likely to provide access compared to bottled water.

Furthermore, clusters with a lower proportion of primary education were 87% less likely [AOR = 0.13, 95% CI: (0.04303, 0.44515)], while those with a higher proportion of middle wealth status were three times more likely (AOR = 3.26, 95% CI: (1.071, 9.9245)) to have access compared to their counterparts (Table 2).

**Table 2:** Multi-level mixed effect logistic regression of availability of observed water, soap, and other cleansing agents for hand washing practice in Ethiopia, 2021 PMA-ET dataset.

Variables	Empty model	Mod one, individual-level characteristics OR (95%CI)	Mode two, community level characteristics OR (95%CI)	Model three, individual and community-level characteristics OR (95%CI)
**Age**				
<25		1.38 (1.0892, 1.7635)		1.38 (1.0891, 1.7631)
25–64		1.46 (1.1440, 1.8638)		1.45 (1.1431, 1.8621)
>65		1		1
**Sex**				
Male		1		1
Female		0.95 (0.85239, 1.0638)		0.95 (0.8529, 1.0643)
**Educational status**				
Not educated		0.60 (0.46875, 0.76818)		0.60 (0. 47191, 0. 77317)
Primary		0.61 (0.46307, 0.79771)		0.61 (0. 46526, 0. 80124)
Secondary		1.01 (0. 796, 2.538)		1.01 (0. 796, 2.538)
Higher		1		1
**Wealth status**				
Poor		0.29 (0.24071, 0.35800)		0.30 (0. 24955, 0.37165)
Middle		0.36 (0.30606, 0.43732)		0.37 (0.31465, 0. 44973)
Rich		1		1
**Media exposure**				
No		1		1
Yes		2.91 (2.5833, 3.2952)		2.88 (2.5565, 3.2615)
**Water source**				
Piped water		0.22 (0.16307, 0.31843)		0.23 (0.16624, 0.32432)
tube well		0.25 (0.15730, 0.42027)		0.28 (0.17372, 0.46466)
dug well		0.30 (0.18721, 0.50669)		0.33 (0.2033, 0.55029)
Spring		0.21 (0.14180, 0.31314)		0.22 (0.14968, 0.33064)
Rainwater		0.09 (0.02054, 0.45972)		0.09 (0.02101, 0.47322)
surface water		0.15 (0.08023, 0.30023)		0.16 (0.08721, 0.3295)
Bottled water		1		1
**Family size**				
<=5		0.93 (0.8437, 1.0384)		0.93 (0.83940, 1.0330)
>5		1		1
**Region**				
Emerging regions			1.88 (0.77976, 4.5391)	1.82 (0.73258, 4.5554)
Developed regions			1	1
**Residence**				
Urban			1	1
Rural			0.38 (0.10333, 1.4126)	0.74 (0.19418, 2.8324)
**Community educational status**				
A lower proportion of primary education			0.09 (0.03038, 0.29978)	0.13 (0.04303, 0.44515)
A higher proportion of primary education			1	1
**Community wealth status**				
A lower proportion of middle-wealth status			1	1
A higher proportion of middle-wealth status			4.11 (1.3831, 12.235)	3.26 (1.071, 9.9245)
**Community media exposure**				
A lower proportion of media exposure				1
A higher proportion of media exposure			3.88 (1.2382, 12.199)	1.81 (0.56696, 5.7942)

#### Random effect (a measure of variation)

The cluster’s correlation coefficient reveals that 88.4% of the variability in the availability of observed water, soap, and other cleansing agents for hand-washing Practice is associated with community-level factors. The overall model accounts for approximately 32.7% of the variation in the availability of observed water, soap, and other cleansing agents for hand washing practice within clusters. The availability of observed water, soap, and other cleansing agents for hand washing Practice MOR in the initial model is 65.0, signifying significant differences (clustering) among communities. Upon incorporating all factors into the model, the unexplained community variance in the availability of observed water, soap, and other cleansing agents for hand washing Practice decreases to a MOR of 43.7 (Table 3).

**Table 3:** random effect model (measure of variation) for availability of observed water, soap, and other cleansing agents for hand washing practice using multilevel logistic regression analysis.

Random effects (Measure of variation for intention to COVID-19 vaccination)	Model 0	Model 1	Model 2	Model 3
Variance	25.14	18.88	16.67	16.91
ICC (%)	88.4	85.2	83.5	83.7
Explained variance (PCV) (%)	Reference	24.9	33.7	32.7
MOR (95% CI)	65.00	48.8	43.1	43.7
**Model fit statistics**				
AIC	12813.4	12014.36	12766.68	11999.74
BIC	12829.65	12160.62	12823.55	12186.62

ICC, intra-class correlation; PCV, proportion change in variance; MOR, median odds ratio; CI, confidence interval; AIC, Akaike’s information criterion; BIC, Schwarz’s Bayesian information criteria.

**Model fit statistics**: the values of AIC and BIC showed subsequent reduction which indicates each model represents a significant improvement over the previous model and it points to the goodness of fit of the final model built in the analysis. Therefore, the final (combined) model that incorporated individual- and community-level factors was selected for the availability of observed water, soap, and other cleansing agents for hand washing practice in Ethiopia (Table 3).

## Discussion

This study aimed to assess individual and community-level factors of availability of observed water, soap, and other cleansing agents for hand washing practices in Ethiopia. In this study participants aged below 25 and between 25 and 64 were significantly associated availability of observed water, soap, and other cleansing agents for hand washing Practice in Ethiopia. This aligns with similar research conducted in various countries. Studies in the United States, Canada, and Australia have also shown a higher likelihood of younger age groups having access to water, soap, and cleansing agents for handwashing compared to older individuals (17, 18). This consistency across different countries reinforces the importance of ensuring equitable access to essential hygiene resources across all age demographics to promote effective hand hygiene practices.

This study also individuals with no formal education and those with only primary education were significantly associated with factors of availability of observed water, soap, and other cleansing agents for hand washing practice. Similar findings regarding the association between education level and the availability of handwashing resourcesa study in Nigeria (19) highlighted that individuals with lower education levels were less likely to have access to adequate handwashing facilities. Similarly, research conducted in India (20) found that individuals with lower levels of education were at a disadvantage in terms of accessing necessary resources for hand hygiene. Contrarily, a study in the United States demonstrated that education level did not significantly influence the availability of handwashing resources, suggesting potential contextual variation (21). Nonetheless, our study echoes the consensus that socioeconomic factors, particularly education, play a crucial role in determining access to hand hygiene resources, emphasizing the need for targeted interventions to bridge these disparities.

Likewise, those who were poor and had middle wealth status were less likely to availability of observed water, soap, and other cleansing agents for hand washing Practice. It reveals a consistent trend regarding the association between wealth status and access to handwashing resources. For example, a study conducted in Bangladesh (22) demonstrated that individuals from lower socioeconomic backgrounds faced greater challenges in accessing handwashing facilities. Similarly, research in Kenya revealed that households with lower wealth status were less likely to have sufficient handwashing resources. Conversely, a study in China suggested that wealth status did not significantly influence access to hand hygiene resources, highlighting potential contextual differences. Nevertheless, our study aligns with the consensus that individuals with poorer or middle-wealth status encounter barriers in accessing adequate handwashing facilities, emphasizing the urgent need for targeted interventions to address these disparities.

In addition, the odds of availability of observed water, soap, and other cleansing agents for hand washing Practice in Ethiopia who had media exposure were more likely as compared with those who had no media exposure. Comparing our findings with previous studies conducted in diverse countries highlights a consistent pattern regarding the influence of media exposure on the availability of handwashing resources. For instance, research conducted in Uganda illustrated that individuals with media exposure were more likely to have access to handwashing facilities (23). Similarly, a study in India indicated that media exposure played a significant role in promoting hygiene practices, including handwashing (24). Media exposure can enhance awareness through public health campaigns, disseminate information on proper handwashing techniques, and influence social norms, thereby promoting hand hygiene practices as a cultural norm supported by educational content and reminders.

Furthermore, water sources, various sources like Piped Water, tube wells, dug wells, springs, rainwater, and surface water were less likely to provide access compared to bottled water. Bottled water’s stringent quality control ensures safer, more reliable access, highlighting socio-economic disparities and infrastructure differences in community water sources, thus providing insights into improving equitable access to safe drinking water.

## Conclusion

The study revealed that both individual and community-level factors were factors of availability of observed water, soap, and other cleansing agents for hand washing practices in Ethiopia. At the individual level, age, educational status, wealth status, water source, and media exposure were significantly associated with the availability of observed water, soap, and other cleansing agents for hand washing practice. At community-level factors, a lower proportion of primary education and a higher proportion of middle wealth were significantly associated with the availability of observed water, soap, and other cleansing agents for hand washing practice. Efforts should focus on increasing access to water, soap, and cleansing agents, particularly among younger individuals, those with lower educational and wealth statuses, and areas with limited media exposure. Additionally, community-level initiatives should aim to enhance primary education levels and promote equitable wealth distribution to ensure the widespread availability of hand-washing resources. These strategies could contribute to fostering a culture of improved hygiene practices across the country.

## Data availability statement

The raw data supporting the conclusions of this article will be made available by the authors, without undue reservation.

## Ethics statement

Ethical review and approval was not required for the study on human participants in accordance with the local legislation and institutional requirements. Written informed consent from the patients/participants or patients/participants' legal guardian/next of kin was not required to participate in this study in accordance with the national legislation and the institutional requirements.

## Author contributions

NK: Conceptualization, Formal analysis, Investigation, Methodology, Software, Validation, Writing – original draft, Writing – review & editing. AD: Conceptualization, Formal analysis, Methodology, Software, Supervision, Writing – original draft, Writing – review & editing. EK: Conceptualization, Formal analysis, Methodology, Software, Supervision, Validation, Writing – original draft, Writing – review & editing. TA: Conceptualization, Formal analysis, Methodology, Software, Validation, Writing – original draft, Writing – review & editing. ET: Conceptualization, Formal analysis, Methodology, Software, Supervision, Validation, Writing – original draft, Writing – review & editing. OA: Conceptualization, Formal analysis, Methodology, Software, Supervision, Validation, Writing – original draft, Writing – review & editing.
